# Biallelic variants in *SLC4A10* encoding a sodium-dependent bicarbonate transporter lead to a neurodevelopmental disorder

**DOI:** 10.1016/j.gim.2023.101034

**Published:** 2024-03

**Authors:** Reza Maroofian, Mina Zamani, Rauan Kaiyrzhanov, Lutz Liebmann, Ehsan Ghayoor Karimiani, Barbara Vona, Antje K. Huebner, Daniel G. Calame, Vinod K. Misra, Saeid Sadeghian, Reza Azizimalamiri, Mohammad Hasan Mohammadi, Jawaher Zeighami, Sogand Heydaran, Mehran Beiraghi Toosi, Javad Akhondian, Meisam Babaei, Narges Hashemi, Rhonda E. Schnur, Mohnish Suri, Jonas Setzke, Matias Wagner, Theresa Brunet, Christopher M. Grochowski, Lisa Emrick, Wendy K. Chung, Ute A. Hellmich, Miriam Schmidts, James R. Lupski, Hamid Galehdari, Mariasavina Severino, Henry Houlden, Christian A. Hübner

**Affiliations:** 1 Department of Neuromuscular Diseases, UCL Queen Square Institute of Neurology, London, United Kingdom; 2 Department of Biology, Faculty of Science, Shahid Chamran University of Ahvaz, Ahvaz, Iran; 3 Narges Medical Genetics and Prenatal Diagnosis Laboratory, Kianpars, Ahvaz, Iran; 4 Institute of Human Genetics, Jena University Hospital, Friedrich Schiller Universität, Am Klinikum 1, Jena, Germany; 5 Molecular and Clinical Sciences Institute, St. George’s, University of London, Cranmer Terrace, London, United Kingdom; 6 Institute of Human Genetics, University Medical Center Göttingen, Göttingen, Germany; 7 Institute for Auditory Neuroscience and InnerEarLab, University Medical Center Göttingen, Göttingen, Germany; 8 Division of Neurology and Developmental Neuroscience, Department of Pediatrics, Baylor College of Medicine, Houston, TX; 9 Texas Children’s Hospital, Houston, TX; 10 Department of Molecular and Human Genetics, Baylor College of Medicine, Houston, TX; 11 Division of Genetic, Genomic & Metabolic Disorders, Discipline of Pediatrics, College of Medicine, Central Michigan University, Mount Pleasant, MI; 12 Department of Pediatric Neurology, Golestan Medical, Educational, and Research Center, Ahvaz Jundishapur University of Medical Sciences, Ahvaz, Iran; 13 Department of Pediatrics, Zabol University of Medical Sciences, Zabol, Iran; 14 Pediatric Neurology Department, Ghaem Hospital, Mashhad University of Medical Sciences, Mashhad, Iran; 15 Neuroscience Research Center, Mashhad University of Medical Science, Mashhad, Iran; 16 Department of Pediatrics, North Khorasan University of Medical Sciences, Bojnurd, Iran; 17 Department of Pediatrics, School of Medicine, Mashhad University of Medical Sciences, Mashhad, Iran; 18 GeneDx, Gaithersburg, MD; 19 Clinical Genetics Service, Nottingham University Hospitals NHS Trust, Nottingham, United Kingdom; 20 Institute of Human Genetics, Klinikum rechts der Isar, School of Medicine, Technical University of Munich, Munich, Germany; 21 Institute of Neurogenomics, Helmholtz Zentrum München, Neuherberg, Germany; 22 Department of Pediatric Neurology and Developmental Medicine and LMU Center for Children with Medical Complexity, Dr. von Hauner Children's Hospital, LMU Hospital, Ludwig-Maximilians-University, Munich, Germany; 23 Department of Pediatrics, Boston Children’s Hospital, Harvard Medical School, Boston, MA; 24 Friedrich Schiller University Jena, Faculty of Chemistry and Earth Sciences, Institute of Organic Chemistry and Macromolecular Chemistry, Jena, Germany; 25 Center for Biomolecular Magnetic Resonance (BMRZ), Goethe University, Frankfurt, Germany; 26 Cluster of Excellence Balance of the Microverse, Friedrich Schiller University Jena, Jena, Germany; 27 Pediatrics Genetics Division, Center for Pediatrics and Adolescent Medicine, Faculty of Medicine, Freiburg University, Freiburg, Germany; 28 Genome Research Division, Human Genetics Department, Radboud University Medical Center, Nijmegen, The Netherlands; 29 CIBSS-Centre for Integrative Biological Signalling Studies, University of Freiburg, Freiburg, Germany; 30 Human Genome Sequencing Center, Baylor College of Medicine, Houston, TX; 31 Neuroradiology Unit, IRCCS Istituto Giannina Gaslini, Genoa, Italy; 32 Center for Rare Diseases, Jena University Hospital, Jena, Germany

**Keywords:** Bicarbonate transporters, Intracellular pH dynamics, Neurodevelopmental disorders, SLC4A10, Slit ventricles

## Abstract

**Purpose:**

*SLC4A10* encodes a plasma membrane-bound transporter, which mediates Na^+^-dependent HCO_3_^−^ import, thus mediating net acid extrusion. *Slc4a10* knockout mice show collapsed brain ventricles, an increased seizure threshold, mild behavioral abnormalities, impaired vision, and deafness.

**Methods:**

Utilizing exome/genome sequencing in families with undiagnosed neurodevelopmental disorders and international data sharing, 11 patients from 6 independent families with biallelic variants in *SLC4A10* were identified. Clinico-radiological and dysmorphology assessments were conducted. A minigene assay, localization studies, intracellular pH recordings, and protein modeling were performed to study the possible functional consequences of the variant alleles.

**Results:**

The families harbor 8 segregating ultra-rare biallelic *SLC4A10* variants (7 missense and 1 splicing). Phenotypically, patients present with global developmental delay/intellectual disability and central hypotonia, accompanied by variable speech delay, microcephaly, cerebellar ataxia, facial dysmorphism, and infrequently, epilepsy. Neuroimaging features range from some non-specific to distinct neuroradiological findings, including slit ventricles and a peculiar form of bilateral curvilinear nodular heterotopia. In silico analyses showed 6 of 7 missense variants affect evolutionarily conserved residues. Functional analyses supported the pathogenicity of 4 of 7 missense variants.

**Conclusion:**

We provide evidence that pathogenic biallelic *SLC4A10* variants can lead to neurodevelopmental disorders characterized by variable abnormalities of the central nervous system, including altered brain ventricles, thus resembling several features observed in knockout mice.

## Graphical abstract

**Figure:**
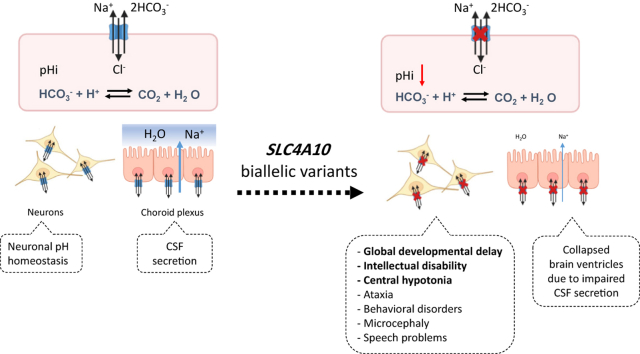

## Introduction

CO_2_, a product of mitochondrial respiration, can freely diffuse across membranes and is controlled by respiration due to its volatility. Because CO_2_ is in equilibrium with HCO_3_^−^ and H^+^, it represents a membrane-permeant acid equivalent. Since HCO_3_^−^ is charged, it cannot move across membranes without facilitation by bicarbonate transport proteins. CO_2_/HCO_3_^−^ is the primary pH buffer of our bodies. The physiological roles of mammalian bicarbonate transporters reflect the chemistry of HCO_3_^−^. They mediate the disposal of waste CO_2_/HCO_3_^−^, the regulation of cellular and systemic pH, and fluid secretion. In mammals, members of the solute carrier (SLC) gene families *SLC4* and *SLC26* have been identified as bicarbonate transporters, many of which are associated with monogenic human diseases, such as distal renal tubular acidosis, hemolytic anemia, corneal dystrophy, chondrodysplasia, chloride diarrhea, and deafness.^1^, ^2^, ^3^

SLC4A10, in particular, utilizes the transmembrane gradient of Na^+^ to drive cellular net uptake of HCO_3_^−^ and thus mediates acid extrusion. Some controversy exists about whether it acts as an electroneutral Na^+^-HCO_3_^−^ cotransporter (NBCn2) or a Na^+^-coupled Cl^−^/HCO_3_^−^ exchanger (NCBE) under physiological conditions.^4^^,^^5^ *Slc4a10* knockout (KO) mice show collapsed brain ventricles, an increased seizure threshold, mild behavioral abnormalities,^6^ impaired vision,^7^ and deafness.^8^ In agreement with the reported phenotypes, *SLC4A10* is abundantly expressed in neurons,^9^ in choroid plexus epithelia,^9^ in the retina,^7^ and in the inner ear fibrocytes.^8^

Although pathogenic variants in several bicarbonate transport proteins are known to cause rare Mendelian human diseases,^1^^,^^10^^,^^11^ variants in *SLC4A10* have hitherto not been associated with any monogenic diseases in humans. Here, we report 6 independent families with 11 individuals affected by a recessive neurodevelopmental disorder (NDD) with variable neuroimaging features. We identified 7 ultra-rare biallelic missense variants and 1 splicing variant in *SLC4A10* in affected individuals, which segregate with the phenotype in the respective family, and provide in silico and functional evidence supporting the pathogenicity for at least 6 variants across 5 families.

## Materials and Methods

### Participant recruitment, data collection, and clinical and genetic investigation

Utilizing proband-only or trio/quad exome/genome sequencing (ES/GS) in families with undiagnosed NDD, screening the genetic databases of several diagnostic and research laboratories worldwide, and using the GeneMatcher data sharing platform,^12^ 6 independent families of Middle Eastern and European ancestries were identified and recruited to the study. Clinical data were uniformly collected using a standardized clinical form. Brain magnetic resonance imaging (MRI) scans and facial images were reviewed by an experienced pediatric neuroradiologist (M.Se.) and dysmorphologist, respectively (M.Su.). Parents and legal guardians of all affected individuals gave their informed consent for the publication of clinical and genetic information according to the Declaration of Helsinki, and the study was approved by the respective local ethics committees. ES or GS and bioinformatics with subsequent candidate variant Sanger sequencing and segregation analysis were carried out on DNA extracted from blood-derived leukocytes at 4 different genetic diagnostic and research laboratories following slightly different protocols.

### Minigene splicing assay

To assess the predicted impact of an intronic variant on splicing, a minigene assay was performed as previously described^13^^,^^14^ with modifications that include the introduction of a c.-8G>T to create a decoy splice acceptor site at position c.-3 as described.^15^ This has been detailed in Supplemental Methods. All primers are shown in Supplemental Table 1.

### Cell culture, localization studies, and intracellular pH recordings

The human *SLC4A10* cDNA was PCR-cloned from a human cDNA library and subcloned into the bidirectional mammalian expression vector pBI-CMV4 (Clontech #PT4443-5). Disease-associated variants were inserted by site-directed mutagenesis and verified by DNA sequencing. N2a cells were cultured at 37 °C/5% CO_2_ with Dulbecco’s Eagle’s Minimum Essential Medium (Gibco #31966-021) supplemented with fetal bovine serum at a final concentration of 10% and 1% penicillin/streptomycin (Gibco #15070063). Undifferentiated N2a cells were transfected with Lipofectamine 3000 (Invitrogen #L3000008) according to the manufacturer’s instructions. We observed transfection rates between 20% and 40% with no obvious differences between different SLC4A10 variants.

For localization studies, N2a cells were fixed with 4% PFA in phosphate-balanced saline (PBS: 40 mM NaCl, 10 mM phosphate buffer, 3 mM KCl, pH 7.4) for 10 min and subsequently washed with PBS. Cells were stained with wheat germ agglutinin (WGA) coupled to Biotin (Biozol #B-1025) at a dilution of 1:500 and a polyclonal rabbit anti-SLC4A10 antibody (1:500) at 4°C overnight. Streptavidin-Alexa Fluor 488 conjugate (1:1000, Invitrogen #S32354) and an Alexa Fluor 546-coupled goat anti-rabbit antibody (1:1000, Invitrogen) served as secondary antibodies.

For pH recordings, transfected cells were loaded with the ratiometric fluorescent dye 2′,7′-Bis(2-carboxyethyl)-5(6)-carboxyfluorescein (BCECF-AM, molecular probes) 48 hours after transfection. Transfected cells were identified by the expression of the fluorescent marker DsRed2. Coverslips with cells were mounted in a 37 °C heated perfusion chamber (Chamlide EC; Live Cell Instruments) of an Axio Observer.Z1 microscope (Zeiss) and superfused with a 5% CO_2_ bicarbonate-buffered solution, containing (in mM) 99 NaCl, 20 Na-gluconate, 5 KCl, 1 MgSO_4_, 1.5 CaCl_2_, 25 NaHCO_3_, and 10 Glucose, at a linear flow rate of 2.8 ml/min. After alternating excitation at 495 nm and 440 nm the emitted light (510-535 nm) was recorded with a CCD-camera (AxioCam MRm; Zeiss). Sodium proton exchange activity was blocked by 5 μM 5-(N-Ethyl-N-isopropyl)-Amiloride (EIPA). Acidification was achieved by replacing 20 mM Na-gluconate with Na-propionate. The recovery (dpH_i_/dt) during acidification, as well as the amplitude of the pH_i_ overshoot after the washout of propionate were analyzed as a measure for acid extrusion. At least 5 coverslips with more than 12 transfected and more than 12 non-transfected cells each were analyzed. The calibration was performed at the end of each experiment with calibration buffers (in mM: 135 KCl, 20 N-methyl-D-glucamine, 4 MgSO_4_, 10 Glucose, 30 HEPES, 10 μM nigericin, and pH ranging from 6.6-7.4). For statistical analysis, we performed a one-way ANOVA followed by Tukey’s posthoc-test.

### In silico structural modeling of the variants

A structural model of human SLC4A10 was predicted using AlphaFold^16^ and visualized using PyMol.^17^ Single nucleotide variants were introduced in silico in PyMol. Figures were generated with PyMol and CorelDRAW Graphics Suite 2022.

## Results

### Identification of *SLC4A10* variants

**Figure 1:**
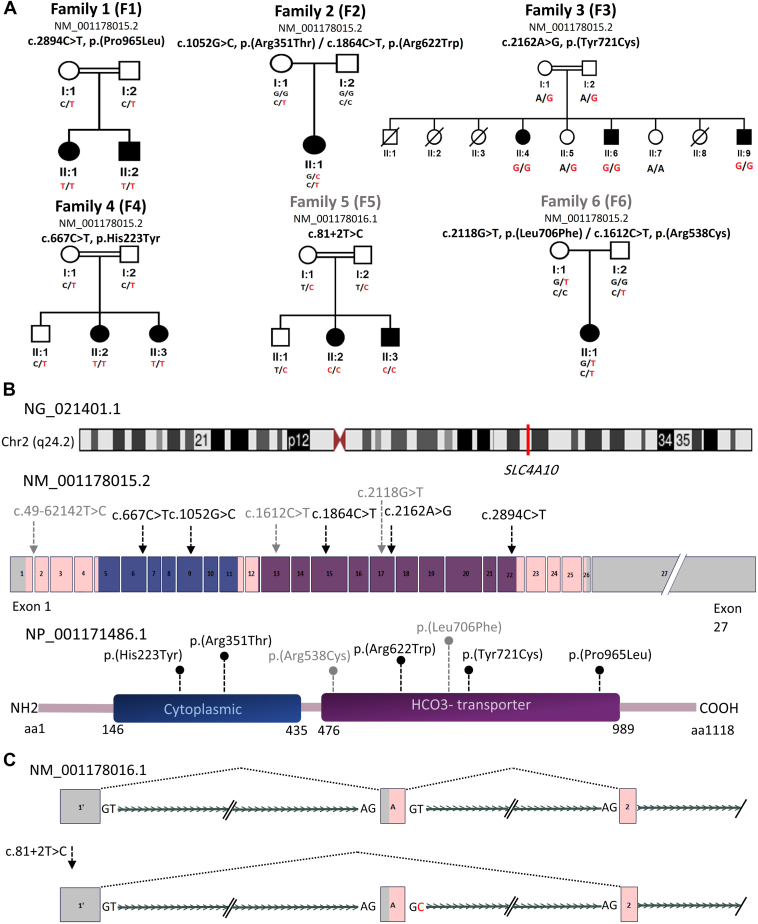
**Pedigrees and genetic characteristics of the *SLC4A10* variants.** A. Pedigrees of families 1-6 showing segregation of the variants within these families. The corresponding genotype is shown under each individual in the pedigrees. B. Chromosome 2 ideogram showing the position of the *SLC4A10* gene (NG_021401.1) at sub-band q24.2 of chromosome 2. Schematic representation of human *SLC4A10* transcript (NM_001178015.2) and protein (NP_001171486.1) indicating the approximate positions of the variants identified in the families. The variants in family 5 and 6 in gray color indicate the uncertainty about their pathogenicity. C. Splicing schematic of the NM_001178016.1: c.81+2T>C variant. The c.81+2T>C variant shows complete skipping of exon 2 for NM_001178016.1 transcript. aa, amino acids.

High-throughput sequencing data analysis targeting currently known genes associated with Mendelian disorders failed to find causative variants explaining the NDD in the families of the current study. Further analysis of the sequencing data and gene/variants led to the identification of 8 ultra-rare variants (7 missense variants and 1 splicing variant) in *SLC4A10* in affected individuals. All identified variants segregated with the disease in the respective families (Figure 1A). All variants are presented based on transcript variant 1 of *SLC4A10* (RefSeq: NM_001178015.2).

Quad-ES, carried out in consanguineous family 1 with 2 similarly affected siblings, identified the homozygous *SLC4A10* variant: c.2894C>T (p.(Pro965Leu)). In family 2, trio-ES revealed that the patient harbors the maternally inherited variant c.1864C>T (p.(Arg622Trp)) and the de novo variant c.1052G>C (p.(Arg351Thr)). To examine phasing, droplet digital polymerase chain reaction was performed and the variants appeared to be in trans, but the long distance between the 2 variants and the possibility of DNA shearing are a limitation of this interpretation (Supplementary Figure 1). ES of probands from 3 other consanguineous families (families 3-5) led to the identification of 3 homozygous variants (family 3: c.2162A>G (p.(Tyr721Cys)), family 4: c.667C>T (p.(His223Tyr)), and family 5: c.49-62142T>C (NM_001178016.1: c.81+2T>C)). Trio-GS in family 6 identified 2 compound heterozygous *SLC4A10* variants: the maternally inherited variant c.2118G>T (p.(Leu706Phe)) and the paternally inherited variant c.1612C>T (p.(Arg538Cys)) (Figure 1B).

Concordant with the reported consanguineous relationships in families F1, F3, F4, and F5, sizeable regions of homozygosity, including stretches of ∼17 Mb (F1:IV5), ∼16 Mb (F3:V4), ∼33 Mb (F4:V2), and ∼17 Mb (F5:V8) encompassing *SLC4A10* were detected (Supplemental Figure 2). All identified variants are either absent or only present at very low allele frequencies (AF) (<0.00012) with no homozygotes in multiple population variant frequency databases with aggregated sequencing data from around 1.5 million individuals (inspected databases are listed in Supplemental Table 2).

In silico analysis and multi-species sequence alignment using various variant prediction tools suggest that the 6 of 7 affected residues are evolutionarily conserved and predicted to be damaging and pathogenic (the computational prediction tools and the respective predictions used for each variant are listed in Supplemental Table 2, and protein sequence alignments of SLC4A10 homologs and locations of the conserved residues and all identified missense variants in this study are represented in Supplemental Figure 3). The genetic characteristics of the *SLC4A10* variants are summarized in Supplemental Table 2. The list of other variants of uncertain significance identified in ES/GS, for which we could not determine their effect, is provided in Supplemental Table 3.

### **Results of minigene splicing assay**

Analysis of the canonical c.81+2T>C variant (identified in family 5) using multiple splice prediction tools suggested a loss of the splice donor site of exon 2 in the third most highly expressed isoform type in the brain as indicated in Genotype-Tissue Expression (GTEx) database (Supplemental Table 4, Supplemental Figure 4B and C, and Supplemental Figure 5). Amplification of transcripts in a minigene assay were consistent with the skipping of exon 2 for the c.81+2T>C variant (Supplemental Figure 4A and D).

### Heterologous expression and intracellular pH recordings

**Figure 2:**
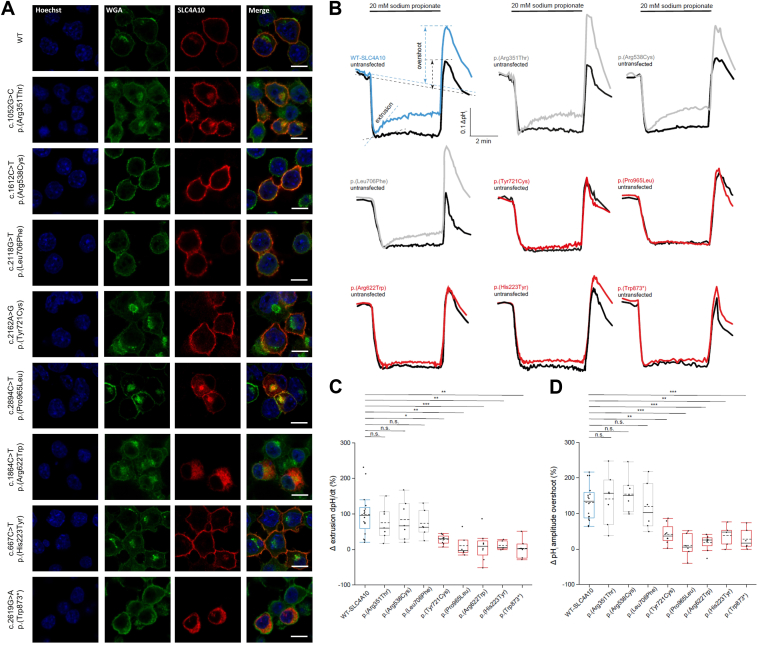
**Cellular localization studies and functional analysis of the variants.** A. Heterologous expression of SLC4A10 WT and SLC4A10 variants in N2a cells. Two days after transfection of N2a cells, cells were fixed and stained with an antibody directed against an N-terminal epitope of SLC4A10 (red) and with the lectin wheat germ agglutinin (WGA, green) to label glycan structures associated with the Golgi apparatus or the plasma membrane. Nuclei were stained with Hoechst 33342. Cells transfected with WT, p.(Arg538Cys), p.(Tyr721Cys), p.(His223Tyr) or p.(Leu706Phe) display a predominant labeling for SLC4A10 at the plasma membrane. Cells transfected with either p.(Pro965Leu), p.(Arg622Trp), or p.(Trp873∗) show a predominant intracellular SLC4A10 labeling. A homozygous loss of function variant, p.(Trp873∗), identified in a patient with NDD, not reported in this study was used as a positive control.^19^ For the variant p.(Arg351Tyr) we found some intracellular labeling apart from the labeling at the plasma membrane. The scale bar represents 10 μm. B. Representative single-cell pH_i_ traces obtained for a cell transfected with either SLC4A10 WT or 1 of the different variants identified (gray refers to variants with undetermined pathogenicity, red to clear loss-of-function variants) in comparison with a trace of an untransfected cell (black) from the same experiment super-fused with bicarbonate buffered solution with 5 μM EIPA to block Na^+^/H^+^ exchange. Cells were acidified by a 5-minute 20 mM sodium propionate pulse. Acid extrusion was determined by a linear fit of the recovery during the first minute after peak acidosis. The overshoot (ie, the alkalinization due to the washout of propionate), which is another readout for the compensatory acid extrusion during the propionate pulse, was calculated by subtracting the baseline pH_i_ from the maximum pH_i_ amplitude after the propionate pulse. Calibration was performed with the high-[K^+^]_o_/nigericin technique. C. The recovery rate upon acidification was calculated and normalized to WT. Compared with WT, it was reduced for the p.(Pro965Leu), p.(Arg351Thr), p.(Tyr721Cys), and p.(His223Tyr) variant. Positive control, p.(Trp873∗), showing diminished extrusion and overshoot in the recordings. D. In accordance, the overshoot amplitudes normalized to WT were reduced as well. Boxplots from 5 independent experiments with more than 60 cells analyzed (the box indicates the q1 (25 %), the q3 (75 %), the whiskers the 1.5 interquartile range, the dashed line the mean and the solid line the median; one-way ANOVA with Tukey’s post-hoc test, n.s.: not significant; ∗*P* < .05; *∗∗P* < .01; ∗∗∗*P* < .001).

To study the functional consequences of the *SLC4A10* variants on acid extrusion, we expressed either the *SLC4A10* wild type (WT) or the different protein variants in undifferentiated N2a cells, a mouse neuroblastoma cell line. Two days post-transfection, the cells were fixed and stained with an antibody directed against the N-terminal epitope of SLC4A10.^6^ The lectin WGA was used to label glycan structures of the plasma membrane.^18^ Cells transfected with the WT construct displayed a strong labeling of the plasma membrane with SLC4A10 and WGA (Figure 2A^19^). Predominant staining of the plasma membrane was also found for Arg538Cys, Tyr721Cys, His223Tyr, or Leu706Phe, whereas Pro965Leu, Arg622Trp, or Trp873∗ showed a predominant intracellular labeling. Signals for the variant Arg351Tyr showed both some intracellular and some labeling of the plasma membrane. Analysis was done with a confocal microscope (LSM 880, Zeiss).

We next loaded cells 2 days after transfection with the ratiometric pH-sensitive dye BCECF and recorded the intracellular pH (pH_i_) in bicarbonate-buffered solutions in the presence of 5 μM EIPA to block Na^+^/H^+^ exchange. pH recordings were done with the Axio Observer.Z1 microscope (Zeiss) and the AxioCam MRm CCD-camera (Zeiss). After an initial baseline recording, cells were acidified by bath application of 20 mM sodium propionate for 5 minutes. Although there was almost no recovery in untransfected N2a cells, cells transfected with the WT construct displayed robust acid extrusion (Figure 2B^19^). The recovery dpH/dt was significantly reduced compared with WT for the variants p.(Pro965Leu), p.(Arg622Trp), p.(Tyr721Cys), and p.(His223Tyr). In agreement, the overshoot upon the termination of the propionate bath application was significantly reduced as well. Notably, the recovery and the overshoot did not differ from WT for the p.(Arg351Thr) variant or the p.(Arg538Cys) and the p.(Leu706Phe) variants (Figure 2B-D^19^).

### Protein modeling

**Figure 3:**
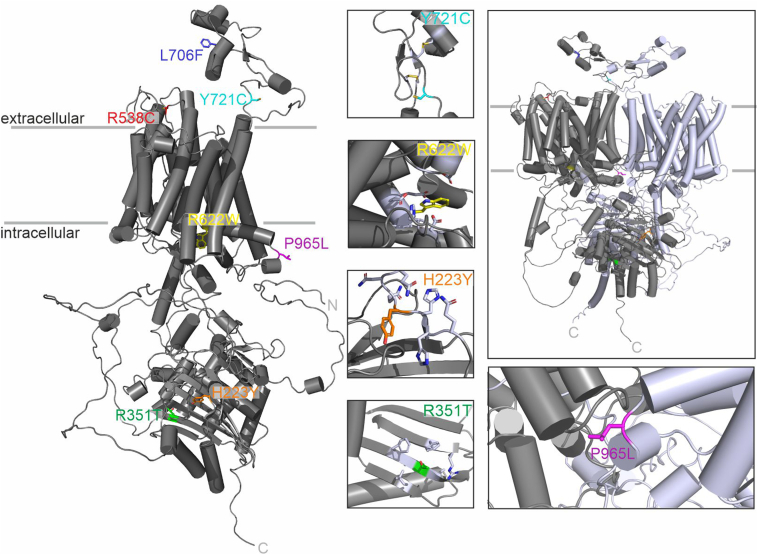
**In silico structural modeling of the variants.** AlphaFold model of human SLC4A10. The location of 7 variants is indicated, and the mutated side chains are shown as sticks. Based on the distribution of hydrophobic residues in the transmembrane helices, the putative position within the membrane is indicated by gray lines. For better visualization, only the monomer is shown on the left. Boxes on the right show zoom-ins of the vicinity of each variant and the model of the homodimeric protein (top right). For clarity, the orientation relative to the main figure may have been altered and different degrees of magnification have been chosen in some cases. Residues deemed relevant for the interaction with the mutated residue are colored in light blue. Atom color code: nitrogen: blue, oxygen: red, and (in zoom for p.(Tyr721Cys)) sulfur: yellow.

To obtain further information about the possible functional consequences of the SLC4A10 variants, we predicted the 3-dimensional structure of SLC4A10 using AlphaFold.^16^ Biochemical and biophysical data indicate that SLC4 transporters are dimers.^20^ Replacement of proline 965 by leucine in the predicted dimer interface of SLC4A10 may compromise protein folding or dimerization because of sterical clashes when the tight proline-mediated turn cannot be formed (Figure 3). Interestingly, a proline residue at the corresponding position in SLC4A8 was seen to be involved in dimer contacts.^21^ For SLC4A10, although previous data suggest that SLC4 monomers function independently,^22^ we cannot exclude that dimerization stimulates monomer function or affects the turnover of the transporter.

Increasing the hydrophobicity may also affect protein-protein interactions. Histidine 223 is found within a stretch rich in other histidine and glutamine residues in the native protein sequence (Supplemental Figure 3). Although usually observed for longer sequence motifs, such polyglutamine stretches have been shown to adopt extended conformations.^23^ Because tyrosine is bulkier than histidine and the pKa values of the respective sidechains differ dramatically, a p.(His223Tyr) substitution may cause steric clashes or alter local protein interactions, further leading to structural rearrangements. Tyrosine 721 locates to an extracellular loop of SLC4A10 in direct vicinity to 4 cysteine residues predicted to form disulfide bridges. By introducing a fifth cysteine into this loop in the disease variant p.(Tyr721Cys), the disulfide bonds may become scrambled, thereby distorting the structure of this loop. Arginine 351 sits in the center of a β-strand within the seven-stranded β-sheet of the SLC4A10 intracellular domain. Here, it is surrounded by large, hydrophobic residues. Structural consequences for replacing this residue with the smaller amino acid threonine (p.(Arg351Thr)) are thus not immediately apparent. However, side-chain interactions with the opposing β-helix may be altered, which could potentially have consequences for cytosolic protein-protein interactions within a cellular context. Finally, replacing arginine 622 with tryptophan (p.(Arg622Trp)) results in the loss of a basic side-chain in a highly acidic pocket. It can be speculated that arginine 622, which is surrounded by 2 aspartic and 3 glutamic acid sidechains, mediates interactions crucial for protein stability and/or function.

In contrast to the previous variants, the alteration of leucine 706 into phenylalanine (p.(Leu706Phe)) is a conservative substitution that is not expected to have an immediate impact on protein structure or function. Also, arginine 538 is a solvent-exposed residue, thus, exchanging it for cysteine (p.(Arg538Cys)) should not affect protein structure dramatically, and no other cysteine residues are found in the vicinity of this variant. However, in both cases, subtle and thus function-affecting consequences to protein folding or dynamics cannot be ruled out.

### Clinical delineation

The cardinal clinical features of the affected individuals in the current cohort are summarized in Table 1 and Supplemental Table 5. Patient 11 has been excluded from clinical statistics due to the patient's clinical phenotype being an outlier, and the pathogenicity of the identified variants remains uncertain. Two persons were born preterm, three had low birth weights, and three cases had congenital microcephaly. Failure to thrive was reported in 3 of 10 persons. All individuals presented with mild-to-severe global developmental delay/intellectual disability. Age at independent walking was delayed by 3.2 ± 2.7 years (range 2-10), and 3 individuals have not yet acquired independent walking by 4 or 7 years of age. Half of the cohort (5/10) remains non-verbal at the median age of 7 years (range 4-37) and another half has mild-to-moderate speech delay.

**Table 1:** Summary of clinical features of affected individuals with biallelic *SLC4A10* variants

Family Number	Family 1		Family 2	Family 3			Family 4		Family 5		Family 6
Affected individual	*P1*	*P2*	*P3*	*P4*	*P5*	*P6*	*P7*	*P8*	*P9*	*P10*	*P11*
Variant type	Missense		Missense	Missense			Missense		Intronic/ Splicing		Missense
Zygosity	Hom		Com. het	Hom			Hom		Hom		Com. het
Variant at the coding sequence (CDS) level (NM_001178015.2)	c.2894C>T		c.1052G>C; c.1864C>T	c.2162A>G			c.667C>T		c.49-62142T>C/ (NM_001178016.1): c.81+2T>C		c.2118G>T; c.1612C>T
Variant at the protein level	p.(Pro965Leu)		p.(Arg351Thr); p.(Arg622Trp)	p.(Tyr721Cys)			p.(His223Tyr)		N/A		p.(Leu706Phe); p.(Arg538Cys)
Sex	M	F	F	F	M	M	F	F	F	M	F
Age at last examination (years)	6	7	7	37	16	33	7	5	4	7	9
Congenital microcephaly	+	+	+	-	-	-	-	-	-	-	N/A
Small birth weight	-	-	+	-	-	-	+	-	+	-	N/A
Failure to thrive	-	-	+	N/A	N/A	N/A	N/A	N/A	+	+	N/A
Microcephaly at present	-	-	+	-	-	+	-	-	+	+	N/A
GDD/ID (Severity)	+	++	+++	+++	+++	+++	++	++	++	++	++++
Non-verbal	-	-	+	+	+	+	-	-	+	-	+
Developmental regression	-	-	+	-	-	-	-	-	-	-	N/A
Dysmorphic features	+	+	+	+	+	+			+	+	N/A
Hearing loss	-	-	-	-	-	-	-	-	-	-	N/A
Impaired vision	-	-	-	-	-	-	-	-	-	-	N/A
Cerebellar ataxia	N/A	N/A	Nap	N/A	N/A	N/A	+	+	N/A	N/A	N/A
Central hypotonia	+	+	+	+	+	+	+	+	+	+	+
Peripheral hypotonia	-	+	+	-	+	-	-	-	+	+	+
Seizures	-	-	Sz-like spells	-	-	-	-	-	-	-	++/DEE
Behavioral/psychiatric problems	AF AP	AF, BP	Anxty	HP	-	-		TT, BP	AF, BP	AF, BP	N/A
Incomplete rotation of the hippocampi	+	+	+	N/A	+	N/A	N/A	+	N/A	+	+
Reduced volume of the frontal horns of the lateral ventricles	+ “Slit-like”	+ “Slit-like”	+ “Slit-like”	N/A	-	N/A	+ (CT scan) Small right frontal horn	-	N/A	+ Small frontal horns	-
A short and dysmorphic corpus callosum	+	+	+	N/A	-	N/A	N/A	-	N/A	-	-
A thin corpus callosum	-	-	-	N/A	-	N/A	N/A	+	N/A	+	+
Peculiar gray matter heterotopia	+	+	+	N/A	-	N/A	N/A	-	N/A	-	-
Other clinical features	-	-	-	-	-	-	-	-	-	-	Choreiform MD

*AF*, autistic features; *AP*, attention problems; *Anxty*, anxiety; *BP*, behavioral problems; *Com.,* compound; *CT*, computerized tomography; *F*, female; *GDD*, global developmental delay; *het*, heterozygous; *Hom*, homozygous; *HP*, happy demeanor; *ID*, intellectual disability; *M*, male; MD, movement disorders; *N/A*, not available or not applicable; *Sz*, seizure; *TT*, temper tantrum.

For the severity: +, mild; ++, moderate; +++, severe.

Upon the most recent physical examination, 4 of 10 individuals had microcephaly. Two of the patients (F2), who acquired speech and walking, were dysarthric with the signs of mild cerebellar ataxia, and gait was broad based in all affected members of F3, who remain nonverbal. Axial hypotonia was uniformly present in all affected individuals, whereas peripheral tone was reduced in only 5 of 10 persons. Spasticity and abnormal deep tendon reflexes were not reported in the patients. Other variable clinical features included strabismus (2/10), autistic features and behavioral problems (5/10), epilepsy (1/10, F1), axial and limb dystonia (F5), developmental regression, and dysphagia requiring G-tube placement in 1 case (1/10, F1). No neurosensory abnormalities were reported in the present series.

**Figure 4:**
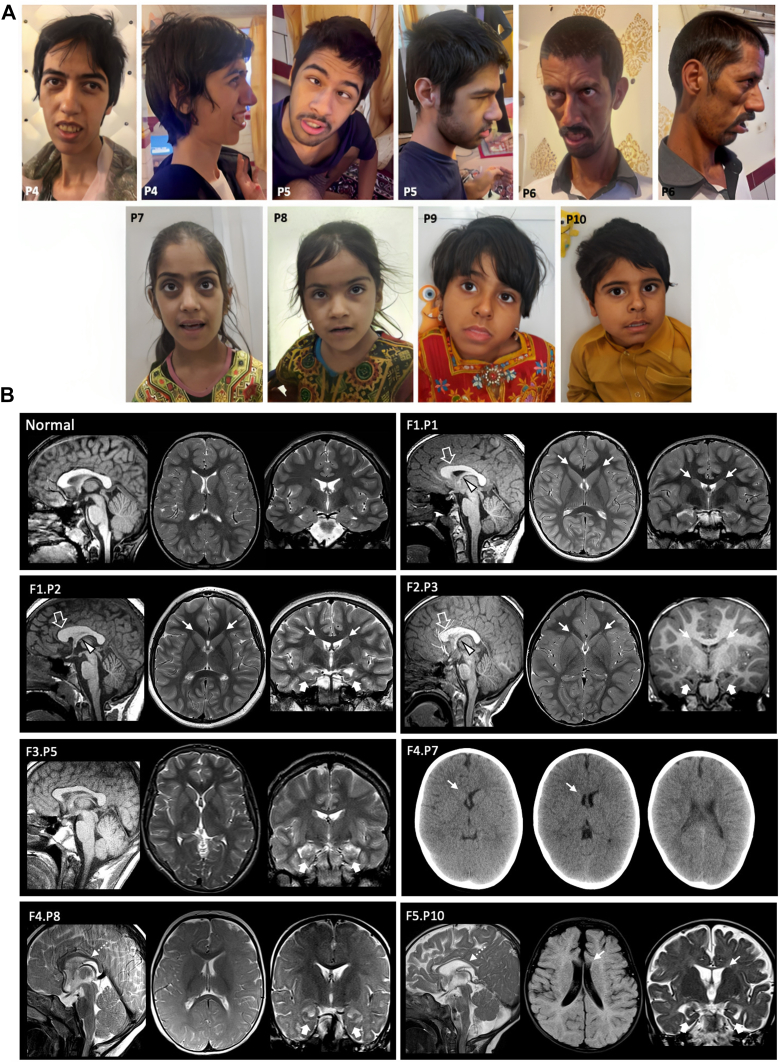
**Summary of the facial and neuroimaging features of the patients with *SLC4A10*-related NDD.** A. P4 has a long and narrow face, bifrontal narrowing, high nasal bridge, medial eyebrow flare, full nasal tip, short philtrum, thin and everted upper lip, full lower lip, and a tall and broad chin. She also has a thin trunk and extremities, long fingers, and long and narrow feet. P5 has a long face, bifrontal narrowing, frontal upsweep of hair, thick eyebrows, right convergent squint, full nasal tip, full-everted upper lip, full-everted lower lip, and a tall and broad chin. He also has small areas of alopecia over the scalp (at the vertex). P6 has a long and narrow face, tall forehead, bifrontal narrowing, mild proptosis, high and narrow nasal bridge, full nasal tip, sunken cheeks, short philtrum, full and everted upper lip, everted lower lip, and a tall chin. He also has a thin trunk and extremities, long fingers, and long and narrow feet. P7 has a long and narrow face, bifrontal narrowing, synophrys, mild proptosis, full nasal tip, and a tall chin. P8 shows a long face, bifrontal narrowing, synophrys, hypertelorism, full nasal tip, and tall and a pointed chin. P9 shows a long face, medial eyebrow flare, synophrys, narrow nasal bridge, full nasal tip, cupid’s bow-shaped upper lip with prominent philtral pillars, full lower lip, and a broad chin. P10 shows low-set eyebrows, synophrys, full nasal tip, prominent philtral pillars, full-everted lower lip, and a broad chin. B. Neuroimaging features of patients with *SLC4A10* variants and of a control subject for comparison. Brain MRI with sagittal T1 or T2 weighted image (first image), axial T2 or fluid attenuated inversion recovery image (middle image) and coronal T2 or T1 weighted image (last image) in subjects P1 at 8 years, P2 at 9 years, P3 at 4 years, P5 at 16 years, P8 at 1.5 years, and P10 at 7 months. Axial head CT images of patient P7 at 3 years. Incomplete rotation of the hippocampi is present in the majority of cases (thick arrows) associated with variable degrees of volume reduction of the frontal horns (thin arrows). Note the unilateral involvement of the frontal horns in P7 and P10. In subjects P1, P2, and P3, there is a short dysmorphic corpus callosum (empty arrows) associated with a small septum pellucidum and short fornices (arrowheads). A thin corpus callosum is noted in subjects P8 and P10 (dotted arrows).

Facial photographs and videos were reviewed for 7 individuals from 3 families (P4-10). No photographs were available for review from P1-3. The most frequent dysmorphic features were full or broad nasal tip (100%), broad, tall, or pointed chin (100%), long face (85.7%), and narrow forehead/bifrontal/bitemporal narrowing (71.4%) as depicted in Figure 4A. Methods for dysmorphology assessment and detailed facial dysmorphic features are presented in Supplemental Tables 6 and 7.

Unlike the other 10 patients exhibiting mainly a neurodevelopmental phenotype, the clinical features of patient 11 from family 6 is more consistent with developmental and epileptic-dyskinetic encephalopathy (the clinical details in the Supplementary Material).

### Neuroimaging Characteristics

Neuroimaging studies were available for review in 7 subjects (8 brain MRI studies in 6 cases and 1 head CT in the remaining subject). The mean age at the last brain MRI or CT was 6.5 years (range 7 months-16 years). The most frequent neuroimaging abnormalities included the incomplete rotation of the hippocampi (6/7, 85.7%) and reduced volume of the frontal horns of the lateral ventricles (5/7, 71.4%). In particular, in 3 cases the frontal horn volume reduction was severe resulting in a “slit-like” configuration, whereas in the other 2 subjects, this finding was more subtle and/or unilateral (Figure 4B). Callosal anomalies were noted in 5/7 cases (71.4%): in 3 persons there was a short and dysmorphic corpus callosum associated with a small septum pellucidum and short fornices; in 2 other cases, there was a thin corpus callosum. In 3 subjects (42.8%), there were multiple small nodules of gray matter heterotopia extending from the temporo-occipital periventricular regions to the temporo-occipital cortex with a peculiar curvilinear disposition (Supplemental Figure 6).

## Discussion

Here, we report the clinical, genetic, and functional evidence obtained from the investigation of 11 patients with biallelic *SLC4A10* variants, initially presenting with an unexplained NDD. The condition is characterized by uniform global developmental delay/intellectual disability with central hypotonia associated with variable speech delay, microcephaly, cerebellar ataxia, facial dysmorphism, as well as epilepsy, but the latter is in only 2 patients. The dysmorphological assessment has not revealed any recognizable facial gestalt. Brain MRI scans revealed non-specific and characteristic neuroimaging findings, including slit-like ventricles and the peculiar form of bilateral curvilinear nodular heterotopia.

The clinical work-up, the neuroradiological evaluation, and the genetic, as well as experimental, data support the causality of the identified *SLC4A10* variants in 7 patients from families 1, 3, and 4. The reduced acid extrusion for variants p.(Pro965Leu) and p.(Arg622Trp) expressed in N2a cells can already be attributed to the reduced or absent localization of the SLC4A10 variants to the plasma membrane. For variants p.(Tyr721Cys) and p.(His223Tyr), preserved localization at the plasma membrane may suggest that the transport activity or transport stoichiometry is affected by these missense variants.

Although the clinical phenotype and the neuroradiological findings fit well with the features observed in the other patients, the functional analysis was less conclusive for 1 of the variants identified in the patient from family 2. Although acid extrusion was diminished compared with WT for the paternally inherited variant p.(Arg622Trp), the maternally inherited variant p.(Arg351Thr) did not differ from WT upon expression of SLC4A10 in N2a cells. Possibly, our in vitro assay lacks sufficient sensitivity or the appropriate cellular context to detect more subtle changes in SLC4A10 transport activity. Another explanation could be that the possible hypomorphic nature of the p.(Arg351Thr) variant only leads to NDD when in compound heterozygosity with a second more deleterious variant, such as p.(Arg622Trp), or in presence of additional genetic modifiers and environmental exposures. As SLC4A10 forms a dimer, p.(Arg622Trp) may act in a dominant-negative manner and trap the p.(Arg351Tyr) variant into an inactive dimeric complex. Future studies will be required to solve this issue. Given the peculiarity and rarity of the neuroimaging features observed in the patients from family 2, which closely resemble the brain MRI findings for both patients from family 1, we nevertheless assume that the phenotype is most likely because of SLC4A10 malfunction.

For the time being, we can also not firmly establish the pathogenicity of the c.81+2T>C variant, identified in family 5, which segregated well within the family. The prediction tools, as well as our minigene assay, suggest an in-frame loss of the first 81 bp of the first coding exon of the transcript ENST00000375514.9/NM_001178016.1 (as is the similar case in 2 other transcripts). According to GTEx, ENST00000375514.9/NM_001178016.1 is 1 of the top 3 most highly expressed isoforms in the human brain (Supplemental Figure 5). An in-frame alternative start codon was not present in the analysis of Kozak similarity scores (data not shown). However, with up to 12 different transcripts annotated in GTEx, the transcript heterogeneity of *SLC4A10* poses a challenge with respect to the functional interpretation. Unlike the second family, the non-specificity of radiological and clinical findings in 2 affected sibs of this family do not add any extra evidence supporting the causality of the variant.

The intracellular pH recordings, as well as the modeling data, suggest that the variants p.(Leu706Phe) and p.(Arg538Cys), found in family 6, may not be functionally relevant. However, we cannot exclude that the variants may result in differences in sensitivity to subtler pH changes or to the regulation by other stimuli. Future experiments should also consider other expression systems, including, eg, choroid plexus cell lines to study potential cell-type-specific effects. Collectively, it appears that the phenotype of this patient, which fits with the diagnosis of developmental and epileptic-dyskinetic encephalopathy, is an outlier in our cohort and is most likely not related to *SLC4A10* variants. However, we cannot confidently rule out the pathogenicity of these 2 variants and the possibility of a blended phenotype in this patient. Although the AF of all the *SLC4A10* variants identified in this study is well below 0.00012, the AF of the variants found in families 5 and 6 are higher than other variants identified in this study, which could add further to uncertainty.

The slit ventricles seen in some patients are a very specific neuroradiological feature that correlates with the observation of collapsed brain ventricles in *Slc4a10* deficient mice. Slc4a10 is abundantly expressed at the basolateral side of choroid plexus epithelial cells.^24^ This epithelial monolayer rests on a highly vascularized connective tissue located at the base of each of the 4 ventricles and is responsible for the secretion of the major part of the cerebrospinal fluid (CSF). It accounts for roughly 500 ml of fluid per day in the adult human.^25^ The molecular mechanisms underlying this choroidal fluid production remain largely elusive. According to the current models, secretion depends on the Na^+^ gradient generated by the Na^+^/K^+^-ATPase that enables the co-transport of ions via Na^+^ coupled transporters to create an osmotic gradient. This osmotic pressure, in turn, drives water secretion through Aquaporin 1 channels. Because brain ventricles are collapsed in *Slc4a10* KO mice and *Slc4a10* is abundantly expressed at the basolateral side of choroid plexus epithelial cells, Slc4a10 is likely the major pathway for basolateral Na^+^ and HCO_3_^−^ entry into choroid plexus epithelial cells. Along this line, deficient basolateral Na^+^ and HCO_3_^−^ uptake will compromise apical Na^+^ secretion. Because it was shown that multiple transporters in the choroid plexus epithelium are mislocalized in *Slc4a10* KO mice,^26^ other factors may contribute to impaired CSF secretion.

Of note, slit-like ventricles are also a recurrent neuroimaging feature in the ultra-rare Chudley-McCullough Syndrome, which is caused by biallelic variants in G protein signaling modulator 2 (GPSM2) (OMIM: 609245).^27^ In this disorder, slit ventricles are associated with early-onset sensorineural hearing loss and a distinctive combination of structural brain abnormalities, whereas psychomotor development is largely preserved. So far, no link between *SLC4A10* and *GPSM2* has been established but may deserve further investigation.

In addition to providing a supportive environment for the brain, instructive CSF-based signaling occurs via the properties of the fluid (eg, flow direction and velocity) and the diverse molecules within the CSF (eg, proteins, neuropeptides, and membrane-bound vesicles) during early brain development.^28^ For example, embryonic CNS progenitors contact the CSF via their apical surface contributing to the retained stemness of these cells.^29^ Indeed, forebrain explants or neural stem cells cultured as neurospheres that are prepared from distinct developmental stages in rodents required age-matched CSF to optimally maintain appropriate progenitor identity, proliferation, and neuronal differentiation.^30^ Thus, compromised CSF production likely affects brain development and may explain the variable structural brain abnormalities in patients. These structural abnormalities in patients with *SLC4A10* loss of function may also explain neurological deficits, such as severe cognitive impairment and autistic behaviors.

Because *SLC4A10* is also expressed in neurons, where it mediates acid extrusion,^6^ *SLC4A10* variants might also profoundly affect neuronal function and network properties and may thus contribute to neurological deficits observed in patients. Synaptic key players, such as glutamate receptors or voltage-gated calcium channels show strong pH dependence, and the effects of pH gradients on synaptic processes are well known. Notably, biallelic variants in *SLC9A1*, another relevant acid extruder, broadly expressed in principal neurons and interneurons,^31^ have also been linked to a rare neurological disorder characterized by progressive cerebellar ataxia with variable sensorineural hearing loss, also known as Lichtenstein-Knorr syndrome (OMIM: 616291).^32^ *Slc9a1* deficient mice display severe epilepsy, although seizures are uncommon among the patients.^33^ Furthermore, SLC4A8 (NDCBE), another Na^+^-dependent HCO_3_^−^ transporter mediating net acid-extrusion, which is closely related to SLC4A10, was shown to play an important role in excitatory transmission.^34^

Although mice and humans with *SLC4A10* loss of function share several features such as cognitive impairment and abnormal brain ventricles, several questions remain when comparing phenotypes. Although *Slc4a10* KO mice display mild impairments of visual acuity and contrast sensitivity in behavioral experiments and altered electroretinograms,^7^ visual deficits are not clear for our patients but can hopefully be clarified by further investigations. Although progressive hearing loss is observed in *Slc4a10* KO mice,^35^ no major hearing loss was noted even for patients older than 30 years of age in our cohort. However, hearing loss at 9 months of age in a mixed C3H background^35^ is opposed to almost complete hearing loss at 2 weeks of age in an almost pure C57BL/6J background,^6^ suggesting that hearing loss due to *SLC4A10* loss of function may depend on genetic modifiers. Another intriguing observation relates to seizure susceptibility upon *SLC4A10* loss of function because seizure susceptibility is decreased in *Slc4a10* KO mice,^6^ which is consistent with the absence or rarity of epilepsy among the patients in this study.

Taken together, we here provide evidence that biallelic variants in *SLC4A10* cause a variable NDD and show the challenge and limitations of establishing new gene-disease association and variant interpretation. Further studies are warranted to clarify additional features and further delineate the phenotypes, as well as developmental and functional mechanisms.

## Data Availability

The data that support the findings of this study are available from the corresponding author upon request.

## Conflict of Interest

James R. Lupski owns stock in 23andMe and is a paid consultant for Genome International. Rhonda E. Schnur is an employee of GeneDx, LLC. The other authors declare no conflicts of interest.
